# Comparing the Structural and Physicochemical Properties of Highland Barley *β*-Glucan from Different Sources: A Focus on Color

**DOI:** 10.3390/foods14020316

**Published:** 2025-01-18

**Authors:** Ping Yu, Xuemin Kang, Pengfei Liu, Zhengzong Wu, Yue Cheng, Bo Cui, Wei Gao

**Affiliations:** 1 Shandong Key Laboratory of Healthy Food Resources Exploration and Creation, School of Food Sciences and Engineering, Qilu University of Technology, Shandong Academy of Sciences, Daxue Road, Changqing District, Jinan 250353, China; chemingmou@163.com (P.Y.); 15689408577@163.com (X.K.); zxliupf@163.com (P.L.); wzzqlu@hotmail.com (Z.W.); cylj144323@163.com (Y.C.); 2 State Key Laboratory of Biobased Material and Green Papermaking, Qilu University of Technology, Shandong Academy of Sciences, Daxue Road, Changqing District, Jinan 250353, China; 3 School of Food Science and Engineering, Qilu University of Technology, Shandong Academy of Sciences, Daxue Road, Changqing District, Jinan 250353, China

**Keywords:** highland barley, *β*-glucan, molecular structure, physicochemical properties

## Abstract

Herein, *β*-glucan (BG) was extracted from different colored varieties of highland barley (HB, *Hordeum vulgare*), defined as BBG, WBG, and LBG depending on the colors of black, white, and blue and their molecular structure and physicochemical properties were investigated through a series of technical methods. The high-performance anion-exchange chromatography (HPAEC) results indicated the extracted BBG, LBG, and WBG mainly comprised glucose regardless of color. The molecular weight (M_w_) of BBG, LBG, and WBG were 55.87 kDa, 65.19 kDa, and 81.59 kDa, respectively. 4-Glc(p), 3-Glc(p), and t-Glc(p) accounted for a larger proportion (>90%) of the total methylated residues according to gas chromatography–mass spectrometry (GC-MS) analysis. Additionally, Fourier transform infrared (FT-IR) spectroscopy revealed that the *β*-linkage of LBG had a greater capacity to develop stronger hydrogen bonds, due to the absence of 3,4-Glc(p). Among them, LBG had a low particle size distribution and a high shear viscosity, showing obvious round aggregates on its surface. Meanwhile, BBG presented a high peak viscosity (PV) and thermal stability. Based on the differences in their molecular structure, it could be concluded that there were different physicochemical properties among BBG, LBG, and WBG.

## 1. Introduction

*β*-glucan is an important natural dietary fiber that is found in plants and microorganisms from natural sources such as grains, fungi, bacteria, and algae [1,2]. The intake of *β*-glucan can increase the viscosity of the digestive fluid in the gastrointestinal tract, extend the emptying time of the stomach, reduce the digestibility of starch, promote insulin secretion, and reduce the absorption rate of glucose, thereby leading to the inhibition and prevention of diabetes. Moreover, *β*-glucan is recognized as a hypocholesterolemic compound that can lower cholesterol and low-density lipoprotein (LDL) levels [3,4,5].

For the past several decades, there has been a general consensus that grain *β*-glucan is made up of mixed linkage (1,3) (1,4) *β*-D-glucose units. Nevertheless, the molecular structure and nutritional efficacy of *β*-glucan from different sources vary remarkably [2,6,7,8,9,10]. In particular, grain-derived *β*-glucan is a kind of non-starch polysaccharide existing in the endosperm and cell wall, which has attracted increasing attention due to its special physiological activity and medicinal value. In a previous study, cereal *β*-glucan showed prebiotic potential via microencapsulation with probiotics, which could provide better protective effects during gastrointestinal digestion [11]. Furthermore, there have been wide applications of cereal *β*-glucan, which can bind to proteins through more exposure sites in protein-based products, resulting in the formation of stronger three-dimensional gel networks and thereby improving the stability of protein gels [12].

The *β*-glucan content of highland barley (HB) is far greater than that of other grain crops [12]. In addition, *β*-glucan extracted from HB can interact with proteins through non-covalent bonds such as hydrogen bonding and electrostatic interactions, leading to the formation of a stronger three-dimensional gel network, which further promotes its application in the food industry [12,13]. The mechanical and barrier properties of highland barley β-glucan (HBBG) film have been enhanced through the incorporation of highland barley prolamin (HBP), resulting in the formation of hydrogen bonds between HBBG and HBP [14]. Both the fine structure and functional characteristics of *β*-glucan are also determined by the species, genetics, and growing conditions of HB [15]. Furthermore, it should be noted that there are many varieties of HB, which can be mainly divided into white, black, and blue according to their color [16]. Li et al. [17] found that the *β*-glucan extracted from the HB core had a higher M_w_ than that extracted from the HB outer bran, showing better foam stability and emulsifying properties. Colored (black and blue) HB varieties showed higher antioxidant, neuroprotective, hypoglycemic, and hypolipidemic properties than white HB [16]. Wang et al. [18] have also reported that colored HB contains a high amount of functional constituents, making it suitable for use in the functional food industry. Therefore, there is no doubt that *β*-glucans extracted from different colored HB varieties exhibit different molecular and physicochemical characteristics. However, limited investigations on the fine structure and physicochemical properties of *β*-glucans extracted from different colored HB varieties have been reported.

The main objective of this study was to extract *β*-glucan from different colored varieties of HB and investigate its molecular structure through a series of technical methods, including high-performance anion-exchange chromatography (HPAEC), size-exclusion chromatography (SEC) equipped with refractive index (RI) and multi-angle laser light-scattering (MALLS) detectors, X-ray diffraction (XRD) and Fourier transform infrared (FT-IR) spectroscopy. In addition, the physicochemical properties of the extracted *β*-glucans were evaluated via scanning electron microscopy (SEM), rapid viscosity analysis (RVA), and thermal stability analysis (TGA). The relevant fluid viscosity results could provide a theoretical basis facilitating the precise processing and high-value utilization (cosmetics and drug delivery) of *β*-glucan.

## 2. Materials and Methods

### 2.1. Materials

HB seeds of different colors (black, white, and blue) were provided by Qinghai Lanke Agriculture and Animal Husbandry Technology Co., Ltd. (Qinghai, China). High temperature resistant α-amylase (BR, 4000 U/g), amyloglucosidase (deriving from *Aspergillus niger*, BR, 100,000 U/g), pancreatin (BR, 4000 U/g), and the dialysis bag (molecular weight cut-off of 3500 Da) were purchased from Yuanye Bio-Technology Co., Ltd. (Shanghai, China). The *β*-glucan Assay Kit (Mixed Linkage) was purchased from Megazyme International LTD, Bray, Co. Wicklow, Ireland. Ethanol (95%, *v*/*v*) was provided by Fuyu Fine Chemical Co., Ltd. (Tianjin, China). Methanol, trifluoroacetic acid, acetic acid, and dichloromethane were purchased from ANPEL Laboratory Technologies (Shanghai) Inc. (Shanghai, China). Monosaccharide standards (fucose, rhamnose, arabinose, galactose, glucose, xylose, mannose, fructose, ribose, galacturonic acid, glucuronic acid, mannuronic acid, and guluronic acid), sodium hydroxide, sodium acetate trihydrate, dimethyl sulfoxide, sodium borodeuteride and acetic anhydride were purchased from Sigma-Aldrich Chemical Co. (St. Louis, MO, USA). Sodium nitrate (AR grade) was purchased from Sinopharm Group Co., Ltd. (Shanghai, China).

### 2.2. Pretreatment of HB Samples

HB was ground using a laboratory mill and then sieved through a 0.50 mm screen to obtain HB flour. The HB flour (50 g) was then suspended in 500 mL of aqueous ethanol and stirred under backflow for 4 h. The mixture was filtered to obtain the precipitate and heated to a constant weight in an oven at 60 °C. The obtained samples were crushed for further analysis.

### 2.3. Extraction and Quantification of β-Glucan

The extraction of *β*-glucan was determined using a previously reported method with minor modifications [11,19]. Please refer to the Supplementary Materials.

**Figure 1:**
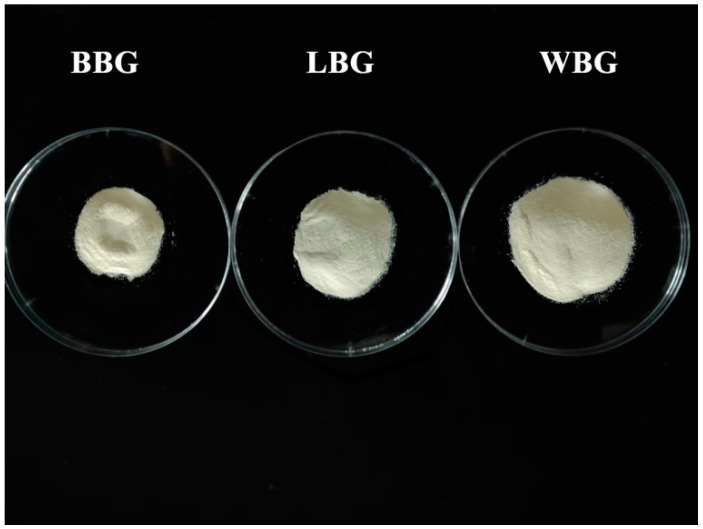
Images of *β*-glucan extracting from different colors of highland barley (BBG indicates black highland barley *β*-glucan; LBG indicates blue highland barley *β*-glucan; WBG indicates white highland barley *β*-glucan).

The *β*-glucan crude extract was defined as BBG, WBG, and LBG, depending on the colors of black, white, and blue, respectively. All extraction processes were repeated three times. Images of the *β*-glucan samples are presented in Figure 1.

### 2.4. Purity, Moisture Content (MC) and Color Variation in Β-Glucan

The purity of BBG, WBG, and LBG was determined using the Congo red method [15,20], while the water content and color variation in samples were analyzed following our reported method [21]. Please refer to the Supplementary Materials.

### 2.5. Molecular Structure Analysis

#### 2.5.1. Monosaccharide Composition

The monosaccharide composition analysis of BBG, WBG, and LBG was determined according to the method described by Bai et al. [19]. Please refer to the Supplementary Materials.

#### 2.5.2. Methylation Analysis

The methylation analysis of BBG, WBG, and LBG was analyzed following the method described by Li et al. [22]. Please refer to the Supplementary Materials.

#### 2.5.3. Molecular Weight Determination

The molecular weight of BBG, WBG, and LBG was measured following the method described by Du et al. [23]. Please refer to the Supplementary Materials.

#### 2.5.4. XRD

The crystalline structure and relative crystallinity of *β*-glucan samples were examined using a previously reported method [24]. Please refer to the Supplementary Materials.

#### 2.5.5. FT-IR

The FT-IR spectra of the *β*-glucan sample were obtained following our previous method [24]. Please refer to the Supplementary Materials.

### 2.6. Particle Morphology Analysis

#### 2.6.1. SEM

The *β*-glucan samples were fixed on a conductive carbon tape and coated with Au/Pd. Subsequently, they were observed through a Hitachi scanning electron microscope (Tokyo, Japan) at a voltage of 5 kV [25].

#### 2.6.2. Particle Size Distribution Determination

The particle size analysis of *β*-glucan was performed using a Microtrac MRB equipped with a dynamic light scattering system (Microtrac Inc., Montgomeryville, PA, USA) with a refractive index of 1.67 and a focusing time of 30 s. At room temperature, the samples were dispersed uniformly into a flowing aqueous cuvette. The test was initiated when the sample concentration reached the standard concentration.

### 2.7. Physicochemical Properties

#### 2.7.1. RVA

The viscosity curve of the *β*-glucan samples was measured following the standard 13 min program [26]. Details can be found in the Supplementary Materials.

#### 2.7.2. Rheological Measurements

The dynamic rheological properties of *β*-glucan were analyzed according to the method of Iqbal et al. [27] with modifications. Details can be found in the Supplementary Materials.

#### 2.7.3. TGA

The thermogravimetric analysis of the *β*-glucan samples was performed according to our previous method [26].

### 2.8. Statistical Analyses

All experimental data were evaluated by running the ANOVA procedure and employing Duncan’s multiple range test (*p* < 0.05).

## 3. Results and Discussion

### 3.1. Characteristics of β-Glucan

The extractability, purity, and MC of BBG, LBG, and WBG are shown in Table 1. The *β*-glucan extractability of black, blue, and white highland barley varieties was 4.94%, 3.32%, and 4.47%, respectively. Moreover, black highland barley exhibited the highest extractability of *β*-glucan. These results correspond with the findings published by Bai et al. [19]. The purity of BBG, LBG, and WBG was all greater than 80%, while the MC values of all the *β*-glucan samples were less than 12%, indicating the extraction method in this study was effective.

The color variation in BBG, LBG, and WBG is shown in Table 2. L* (0 (black) to 100 (white)), a* (−80 (greenness) to 100 (redness)) and b* (−80 (blueness) to 70 (yellowness)) reflect whiteness, redness, and yellowness, respectively, while ΔE indicates the color difference [21]. There were significant differences among all the *β*-glucan samples for b* (ranging from 9.37 to 11.60) and ΔE values, which might be closely related to appearance and consumer acceptance. Interestingly, LBG had the highest L* value of 83.38, indicating that it showed an opaque white color.

### 3.2. Molecular Structure Analysis

#### 3.2.1. Monosaccharide Composition and Methylation Analysis

**Figure 2:**
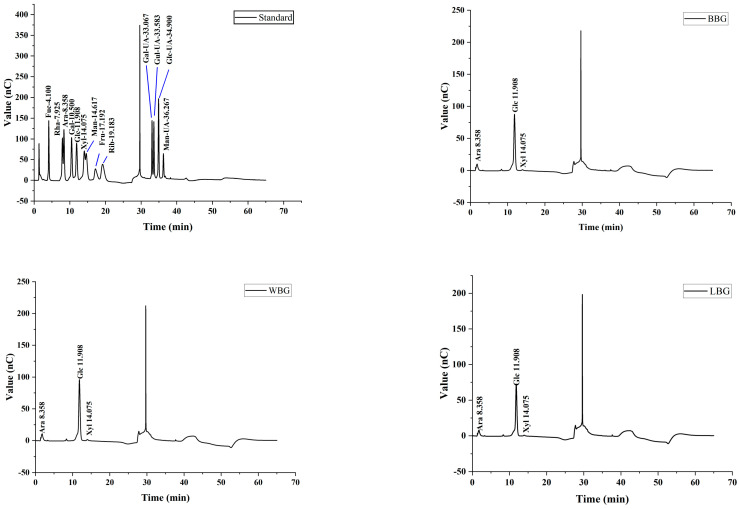
Monosaccharide composition chromatograms of standards, BBG, LBG and WBG samples (BBG indicates black highland barley *β*−glucan; LBG indicates blue highland barley *β*−glucan; WBG indicates white highland barley *β*−glucan).

The monosaccharide composition chromatograms of the standards and the BBG, LBG, and WBG samples are shown in Figure 2. Furthermore, the percentage of the monosaccharides is displayed in Table 3. Monosaccharide standards (fucose, rhamnose, arabinose, galactose, glucose, xylose, mannose, fructose, ribose, galacturonic acid, glucuronic acid, mannuronic acid, and guluronic acid) were employed to identify and measure the corresponding peaks in the monosaccharide composition analysis of different colors of HB. There were three peaks in the elution curves of BBG, LBG, and WBG shown on the standard substance graph, which were related to arabinose, glucose, and xylose. As expected, the extracted BBG, LBG, and WBG mainly comprised glucose, regardless of the color. The other impurities were probably the source of the trace levels of xylose and arabinose [19].

According to Table 4, there were seven different linkage patterns in the GC-MS spectrum of methylated alditol acetates obtained from BBG and WBG. Meanwhile, LBG had six linkage patterns. Among the samples, 4-Glc(p), 3-Glc(p), and t-Glc(p) accounted for a larger proportion (>90%) of the total methylated residues, indicating that the extracted *β*-glucan mainly comprised three forms of sugar linkages. Additionally, there was no 3,4-Glc(p) in LBG.

#### 3.2.2. Molecular Weight Distribution Analysis

**Figure 3:**
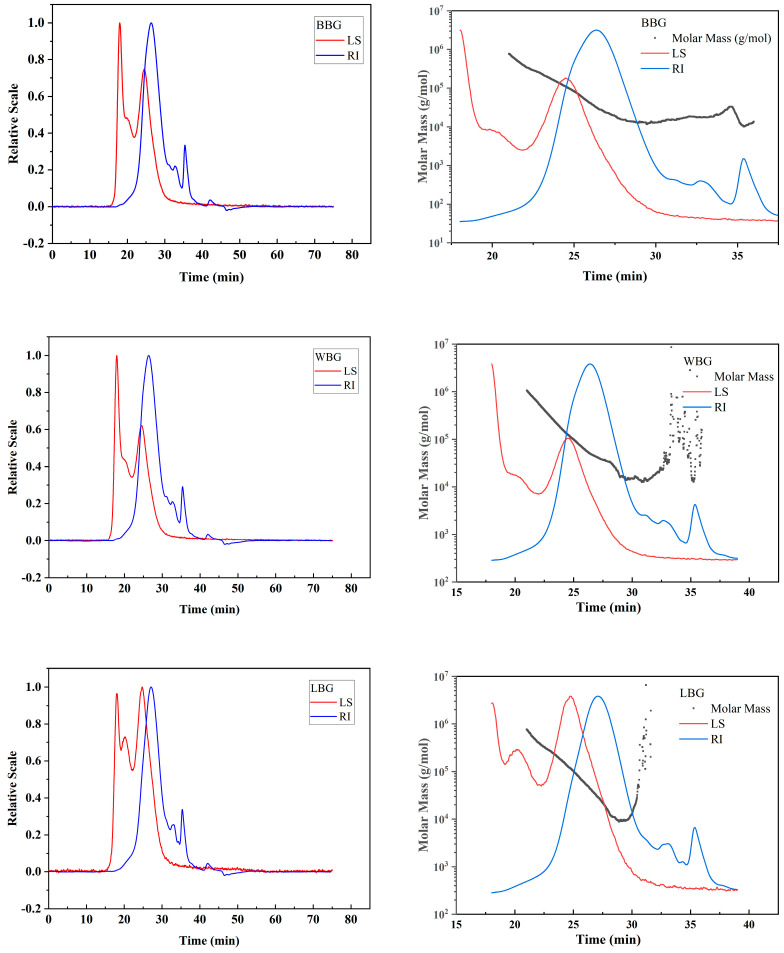
The profiles of molar mass/RI/LS signals versus elution time (min) of BBG, LBG, and WBG (BBG indicates black highland barley *β*-glucan; LBG indicates blue highland barley *β*-glucan; WBG indicates white highland barley *β*-glucan).

The multi-angle laser light scattering (LS), refractive index (RI), and molar mass curves of BBG, LBG, and WBG are presented in Figure 3. There were two peaks that emerged within the elution time from 10 to 30 min in the LS profiles of BBG and WBG, while the LS profiles of LBG exhibited three peaks, suggesting the presence of larger aggregates of *β*-glucan in the NaNO_3_ solution, especially for the LBG sample [28]. Moreover, peak II that emerged at approximately 20 min could be due to the flexible individual chains of *β*-glucan [29]. These findings could demonstrate that the samples were unevenly dispersed in the NaNO_3_ solution through the presence of two forms of larger aggregates and flexible individual chains. The disappearance of peak II in the BBG and WBG samples might be related to the formation of intermolecular interaction among the side chains of *β*-glucan, thus forming a loose network [28].

There were three peaks of RI signal in the BBG, LBG, and WBG curves, but these peaks emerged at different elution times and in different peak areas. In addition, the solvent peak of the mobile phase (NaNO_3_ solution) emerged at nearly 37 min. As shown in Table 5, the molecular weight (M_w_) of BBG was 55.87 kDa, which was lower than that of LBG (65.19 kDa) and WBG (81.59 kDa). It was also previously reported that the M_w_ (648.2 kDa) of *β*-glucan extracted from the inner fraction of HB was higher than that extracted from the HB outer bran (58.59 kDa) [17]. Moreover, the reduced polydispersity (Mw/Mn) values of LBG, BBG, and WBG were 2.83, 2.36, and 2.23, respectively, indicating a more uniform molecule distribution of *β*-glucan [19]. Additionally, the findings illustrated that the *β*-glucan extracted from different colored varieties of highland barley showed different sizes and conformations [30].

#### 3.2.3. XRD Analysis

**Figure 4:**
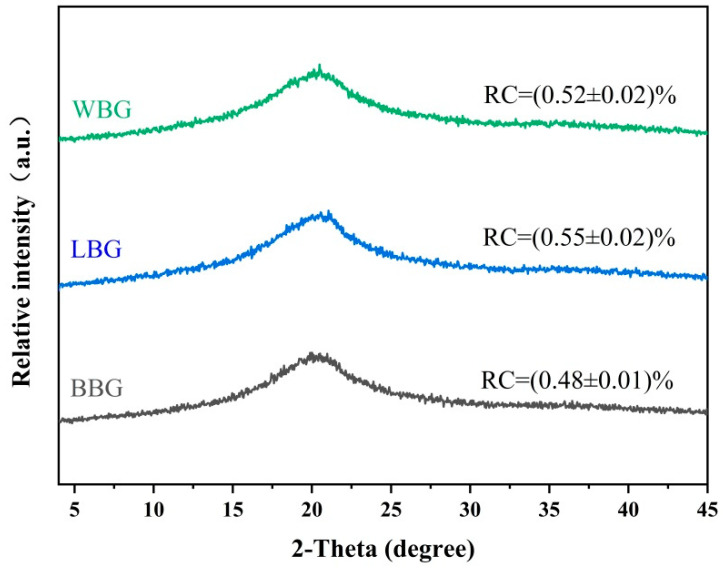
XRD spectrum of BBG, LBG, and WBG (BBG indicates black highland barley *β*-glucan; LBG indicates blue highland barley *β*-glucan; WBG indicates white highland barley *β*-glucan).

The XRD patterns and relative crystallinity (RC) of BBG, LBG, and WBG are shown in Figure 4. It is evident that the diffraction patterns of BBG, WBG, and LBG all exhibited a wide peak at 2θ = 20°, indicating the formation of the relatively regular crystalline and amorphous forms during the extraction and purification process [29]. A similar result was also observed by Bai et al. [19], who reported that there was a broader peak at 2θ = 20° in the crystalline region of *β*-glucan after heat fluidization and microwave treatments. In fact, the crystalline structure of the *β*-glucan samples was significantly affected by the extraction method. The barley *β*-glucan extracted assisting with the high-speed centrifugal vortex method showed a narrower peak than the control sample [31]. Furthermore, new weak peaks at approximately 2θ = 13° were observed for BBG, WBG, and LBG, leading to the conclusion that there were multiple crystal-like structures and lattice types in the crystalline portion [19]. It should be noted that the RC values of all samples ranged from 0.48 to 0.52%, which were much lower than that of hull-less barley *β*-glucan (RC = 28.37%) with a high M_w_ (3100 kDa) [32]. Li et al. [32] concluded that a high molecular weight and a dense micrographic network contributed to the formation of crystalline structures.

#### 3.2.4. FT-IR Analysis

**Figure 5:**
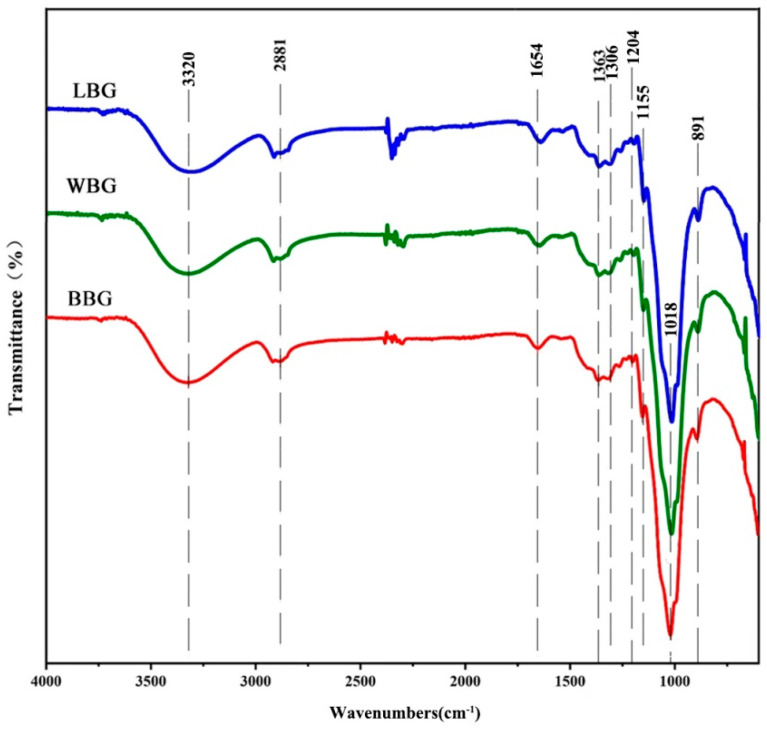
FT-IR spectrum of BBG, LBG, and WBG (BBG indicates black highland barley *β*-glucan; LBG indicates blue highland barley *β*-glucan; WBG indicates white highland barley *β*-glucan).

The FT-IR spectra curves of BBG, LBG, and WBG are shown in Figure 5. All the sample spectra exhibited a similar trend, suggesting that there were little changes in the functional groups of *β*-glucan among the different colored HB varieties. The peaks in the region of 3343–3325 cm^−1^ were due to the asymmetric stretching vibration of hydroxyl O-H resulting from the interactions between glycosidic linkages within the polysaccharide chain [33], while the peaks around 2881 cm^−1^ were attributed to C-H stretching vibration on saturated carbons [34]. Furthermore, it was observed that the characteristic peaks representing hydroxyl O-H of BBG, LBG, and WBG emerged at 3343.3 cm^−1^, 3325.6 cm^−1^, and 3322.7 cm^−1^, respectively. According to the harmonic oscillator model [35], the inter- and intra-molecular hydrogen bonding interactions between hydroxyl groups within the polysaccharide chain were in the following order: WBG > LBG > BBG. Meanwhile, the chemical shift value around 1654.2 cm^−1^ was due to the asymmetric stretching of C=O, which has also been reported to be due to the binding of *β*-glucan to water [36]. Additionally, the vibration peaks between 1017.9 and 1155.4 cm^−1^ were related to the C-O-C stretching of glycosidic linkages [37]. Interestingly, the intensity of these two peaks was in the following order: LBG > WBG > BBG, which indicated that more molecular chains of *β*-glucan were broken depending on the color during the heat extraction processing. This result could also be explained by the harmonic oscillator model mentioned above, which proposes that molecular interaction is enhanced with a decrease in the peak frequency. Furthermore, the absorption peak at approximately 893.6 cm^−1^ was associated with the stretching vibrations of the *β*-linked glycosidic bond [19,38]. Due to the absence of 3,4-Glc(p), the strongest intensity of this characteristic “anomeric region” was observed in LBG, indicating that the *β*-linkage of pyranose obtained from blue highland barley had more opportunity to form stronger hydrogen bonds [39,40].

### 3.3. Particle Morphology Analysis of β-Glucan

#### 3.3.1. Morphology Observation

**Figure 6:**
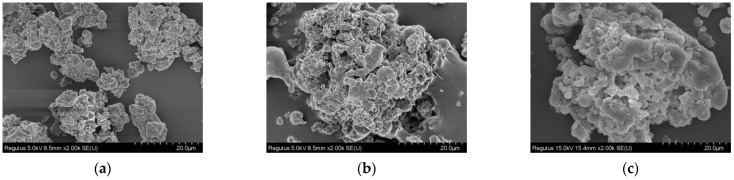
Surface morphology of BBG (**a**), LBG (**b**), WBG (**c**) with a magnification of 2000 (BBG indicates black highland barley *β*-glucan; LBG indicates blue highland barley *β*-glucan; WBG indicates white highland barley *β*-glucan).

The SEM morphologies of BBG, LBG, and WBG are shown in Figure 6. Generally, all the *β*-glucan samples exhibited rough surfaces, which showed spheroidal aggregates of different sizes. This may be due to the greater molecular weight of *β*-glucans extracted by means of hot water without seriously damaging its molecular structure. Similar results have been proposed by Dong et al. [37], who found that thermal processing (steaming or microwave) could hinder the aggregation of oat *β*-glucan. As shown in Figure 6, it can also be concluded that there were many obvious round aggregates in LBG, while WBG and BBG were mostly composed of large and compact aggregates. Cory et al. [41] suggested that it may also be related to the growing environment and gene regulation. Ma et al. [42] reported that there were compact aggregated clusters with honeycomb-like structures in yeast *β*-glucan.

#### 3.3.2. Particle Size Distribution Determination

**Figure 7:**
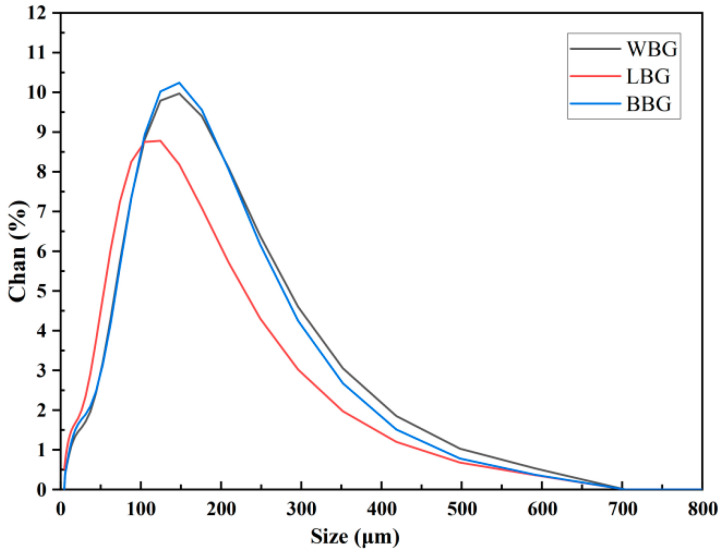
Particle-size distribution of BBG, LBG, and WBG (BBG indicates black highland barley *β*-glucan; LBG indicates blue highland barley *β*-glucan; WBG indicates white highland barley *β*-glucan).

The particle size distribution curves and parameters (D(4,3), D(3,2), D_10_, D_50,_ and D_90_) of the BBG, WBG, and LBG samples are shown in Figure 7 and Table 6, respectively. Particle size distribution is an important factor affecting the aggregation state and conformation of *β*-glucan [43]. Specifically, the D_50_ values of WBG, BBG, and LBG were 112.75 μm, 112.15 μm, and 87.93 μm, respectively. The D(4,3) values of WBG, BBG, and LBG were in descending order with 130.60 μm, 127.75 μm, and 107.70 μm, respectively. Meanwhile, the WBG, BBG, and LBG samples exhibited D(3,2) values of 55.94 μm, 56.57 μm, and 40.72 μm, respectively. The closer the values of D(3,2) and D(4,3), the more regular the shape of the sample particles; this means that the particle size distribution is more concentrated [44]. These results indicated that LBG provided the best dispersion in water, which was similar to the finding observed by SEM.

### 3.4. Physicochemical Properties

#### 3.4.1. Rapid Viscosity Analysis (RVA)

**Figure 8:**
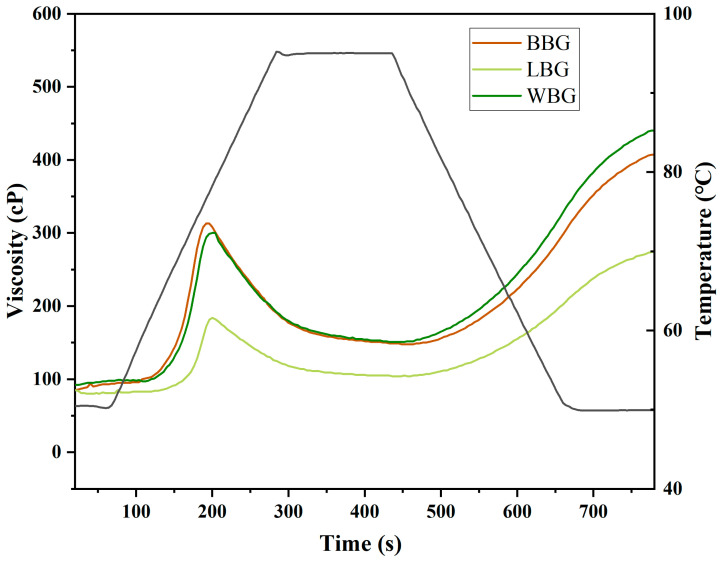
Rapid viscosity analysis of BBG, LBG, and WBG (BBG indicates black highland barley *β*-glucan; LBG indicates blue highland barley *β*-glucan; WBG indicates white highland barley *β*-glucan).

The gelatinization performance of BBG, LBG, and WBG measured by RVA is shown in Figure 8. Meanwhile, the pasting parameters (pasting temperature, PT; peak viscosity, PV; trough viscosity, TV; final viscosity, FV; breakdown, BD; and setback, SB), reflecting the molecular structure evolution of *β*-glucan during the heating cycle, are presented in Table 7. The BBG and WBG samples displayed similar pasting profile shapes, which were distinctly different from that of LBG, thus indicating a significant change in their pasting parameters. Specifically, the highest PV value of 296.5 cP was observed in BBG, followed by WBG (295 cP) and LBG (183 cP). As shown in the thermal stability results, LBG showed the strongest water-binding capacity among all the *β*-glucan samples, thus exhibiting a poor ability to form a network gel structure and a low PV value during the heating cycle. Correspondingly, the ability of the sample to swell and bind water was positively correlated with the gelatinization temperature [45]. Therefore, the PT values of BBG, WBG, and LBG were in the descending order of 73.38 °C, 72.95 °C, and 70.18 °C, respectively. Furthermore, the stability and degradation of the *β*-glucan paste during the heating–cooling cycle are reflected by the BD and SB values [46]. As shown in Table 7, LBG had the lowest BD and SB values of 78.5 and 170.5, respectively, indicating that the stability of LBG paste was higher than that of BBG and WBG pastes. Moreover, the paste viscosity was positively influenced by particle size [47]. These observations were also consistent with the particle size distribution results.

#### 3.4.2. Rheological Measurements

**Figure 9:**
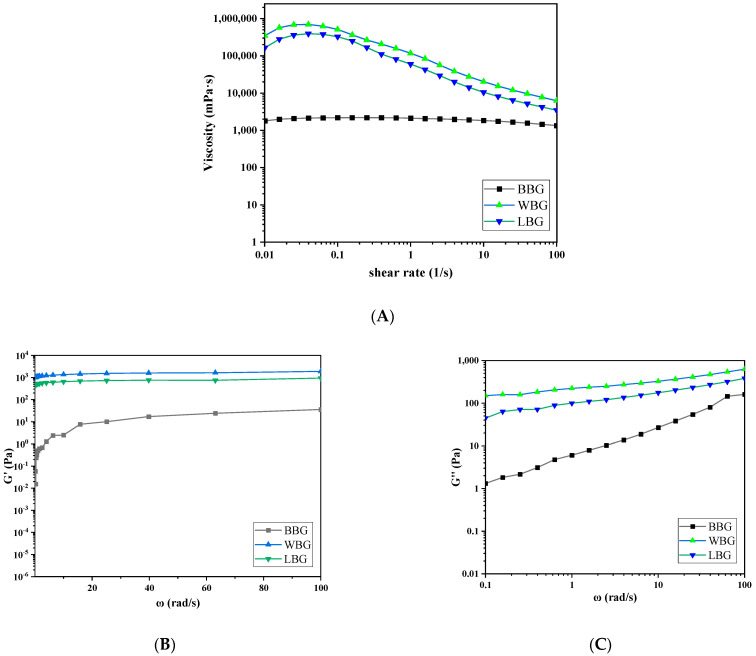
Apparent viscosity (**A**) and dynamic rheological (**B**,**C**) curves of BBG, WBG, LBG (BBG indicates black highland barley *β*-glucan; LBG indicates blue highland barley *β*-glucan; WBG indicates white highland barley *β*-glucan).

The dynamic rheological data of BBG, WBG, and LBG are shown in Figure 9. The inter-chain relationships between polysaccharides can be understood through the observation of rheological behavior. *β*-glucan with a 4% concentration mainly manifests as intermolecular entanglement and gel formation in an aqueous solution [39]. In dynamic rheological behavior analysis, the storage modulus (G′) characterizes the strength of the gel and the loss modulus (G″) indicates the change in the viscosity of the sol, which could be employed to investigate the viscoelastic properties of mixed gel systems. The G′ values of WBG and LBG were higher than the G″ values, indicating that their fluids showed a weak gel structure [48]. Interestingly, the G′ value of the BBG fluid was lower than the G″ value, which is a typical characteristic of an entangled viscoelastic liquid, indicating that it could be used in jam food systems with high viscosity maintenance requirements. In addition, both WBG and LBG had higher dynamic modulus (G′ and G″ values) than BBG, suggesting stronger viscoelastic behaviors. The gelation process of grain *β*-glucan was found to depend on the molar mass and aggregation state of *β*-glucan molecules.

As shown in Figure 9, the viscosity of WBG and LBG decreased significantly with increasing shear rate, exhibiting shear-thinning flow behavior, while the viscosity of BBG remained stable. Li et al. [49] concluded that this phenomenon was due to the same directional movement of *β*-glucan molecules and the destruction of molecular entanglement caused by strong shear action. The shear viscosity of *β*-glucan dispersion was related to the average molecular weight, concentration, and composition [50]. The WBG and LBG samples with a large molecular weight showed a higher shear viscosity than the BBG sample. This may be due to the stronger intermolecular interactions and the higher entanglement among molecular chains in the WBG and LBG samples with a high M_w_. Similarly, *β*-glucan extracted from the HB core with a high M_w_ exhibited higher apparent viscosity than that extracted from the outer bran due to the stronger molecular chain entanglements [17]. Furthermore, the flow behavior index (n) of the different samples was less than 1, indicating that all samples were pseudoplastic fluids. The gel-like properties of high-molecular-weight *β*-glucan suggest potential applications in food processing, cosmetics, and drug delivery [51].

#### 3.4.3. Thermal Property Measurement

**Figure 10:**
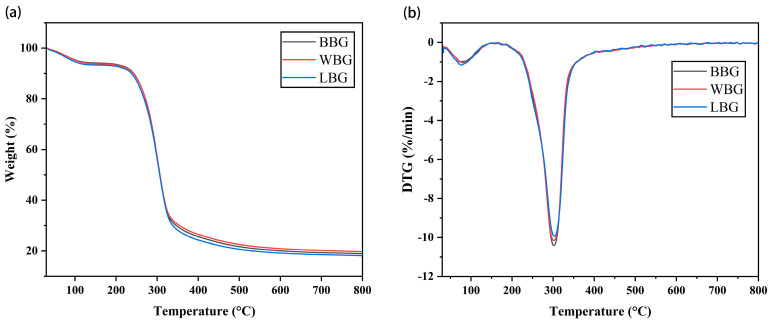
TG (**a**) and DTG (**b**) curves of BBG, LBG, WBG (BBG indicates black highland barley *β*−glucan; LBG indicates blue highland barley *β*−glucan; WBG indicates white highland barley *β*−glucan).

The thermogravimetric (TG) and differential thermogravimetric (DTG) spectra of BBG, LBG, and WBG are shown in Figure 10. There were two distinct stages of weight loss shown in the curves of all samples. The DTG curve was derived from TGA, representing the rate of weight loss. The initial weight loss was observed between 50 °C and 130 °C, which was related to the release of water from the samples [52]. More interestingly, the first maximum mass loss rates were observed in the BBG, LBG, and WBG samples at temperatures of 75.7 °C, 84.6 °C, and 75.4 °C, respectively, revealing that LBG had the strongest water-binding capacity. In a previous study, it was also reported that the stable weight loss of *β*-glucan at high temperatures (<200 °C) was caused by non-covalent bond breaking [53]. Meanwhile, the significant weight loss occurring in the 180–350 °C range was attributed to the decomposition of *β*-glucan. The shapes of the DTG curves for BBG, LBG, and WBG exhibited narrow peaks, indicating that all the samples had a more uniform molecular weight distribution [31]. As expected, the pyrolysis temperatures of the three BG samples almost overlapped, suggesting that there was little difference in the molecular structure among the different colored samples. Specifically, as illustrated in Figure 9B, the highest pyrolysis temperature was found in LBG (303.3 °C) followed by WBG (301.9 °C) and BBG (301.7 °C).

## 4. Conclusions

Our present work showed that HB *β*-glucan mainly comprised glucose, regardless of the color. However, there were still many differences in the molecular structure parameters of the BBG, LBG, and WBG samples. 4-Glc(p), 3-Glc(p), and t-Glc(p) accounted for a larger proportion (>90%) of the total methylated residues. Additionally, there was no 3,4-Glc(p) in LBG. The M_w_ values of *β*-glucan extracted from the different colored HB varieties were in the following order: WBG > LBG > BBG, which resulted in different physicochemical properties for BBG, LBG, and WBG. Specifically, LBG presented the best dispersion in water and the highest pyrolysis temperature. Meanwhile, WBG and LBG samples, with large molecular weight, showed a higher shear viscosity than the BBG sample. Interestingly, the G′ value of the BBG fluids was lower than the G″ value, which is a typical characteristic of entangled viscoelastic liquids. In summary, the gel-like properties of high-molecular-weight *β*-glucan suggest potential applications in food processing, cosmetics, and drug delivery.

## Figures and Tables

**Table 1:** Extractability, purity, and MC of BBG, LBG, and WBG (BBG indicates black highland barley *β*-glucan; LBG indicates blue highland barley *β*-glucan; WBG indicates white highland barley *β*-glucan).

Sample	Extractability/%	Purity/%	MC (%)
BBG	4.94 ± 0.09 ^b^	89.06 ± 1.99 ^b^	10.91 ± 0.29 ^a^
WBG	4.47 ± 0.18 ^b^	82.37 ± 1.23 ^a^	11.84 ± 0.09 ^b^
LBG	3.32 ± 0.24 ^a^	83.69 ± 1.74 ^a^	11.85 ± 0.22 ^b^

The values correspond to the mean ± standard deviation (*n* = 3). Different letters in the same column indicate significant differences (*p* < 0.05).

**Table 2:** Color variation in BBG, LBG, and WBG (BBG indicates black highland barley *β*-glucan; LBG indicates blue highland barley *β*-glucan; WBG indicates white highland barley *β*-glucan).

Sample	L*	a*	b*	ΔE*
BBG	72.40 ± 0.07 ^b^	1.94 ± 0.01 ^ab^	10.72 ± 0.01 ^c^	17.31 ± 0.08 ^a^
WBG	82.36 ± 1.76 ^a^	3.29 ± 0.75 ^a^	11.60 ± 1.05 ^a^	7.11 ± 0.16 ^c^
LBG	83.38 ± 0.01 ^a^	1.41 ± 0.04 ^b^	9.37 ± 0.09 ^b^	6.25 ± 0.01 ^b^

The values correspond to the mean ± standard deviation (*n* = 3). Different letters in the same column indicate significant differences (*p* < 0.05).

**Table 3:** Monosaccharide composition (molar ratio/%) of BBG, WBG, and LBG (BBG indicates black highland barley *β*-glucan; LBG indicates blue highland barley *β*-glucan; WBG indicates white highland barley *β*-glucan).

Sample	Arabinose	Glucose	Xylose
BBG	1.47%	96.75%	1.78%
WBG	1.52%	96.65%	1.83%
LBG	1.71%	95.97%	2.32%

**Table 4:** Methylation analysis of BBG, LBG, and WBG (BBG indicates black highland barley *β*-glucan; LBG indicates blue highland barley *β*-glucan; WBG indicates white highland barley *β*-glucan).

Sample	Linkage Patterns	Derivative Name	RT	Molar Ratios (%)
BBG	t-Ara(f)	1,4-di-O-acetyl-2,3,5-tri-O-methyl arabinitol	6.001	1.61
	t-Glc(p)	1,5-di-O-acetyl-2,3,4,6-tetra-O-methyl glucitol	8.800	6.97
	4-Xyl(p)	1,4,5-tri-O-acetyl-2,3-di-O-methyl xylitol	11.409	1.25
	3-Glc(p)	1,3,5-tri-O-acetyl-2,4,6-tri-O-methyl glucitol	11.976	17.37
	4-Glc(p)	1,4,5-tri-O-acetyl-2,3,6-tri-O-methyl glucitol	13.918	66.39
	3,4-Glc(p)	1,3,4,5-tetra-O-acetyl-2,6-di-O-methyl glucitol	16.064	1.42
	4,6-Glc(p)	1,4,5,6-tetra-O-acetyl-2,3-di-O-methyl glucitol	18.153	4.99
WBG	t-Ara(f)	1,4-di-O-acetyl-2,3,5-tri-O-methyl arabinitol	5.987	1.59
	t-Glc(p)	1,5-di-O-acetyl-2,3,4,6-tetra-O-methyl glucitol	8.779	6.19
	4-Xyl(p)	1,4,5-tri-O-acetyl-2,3-di-O-methyl xylitol	11.391	1.30
	3-Glc(p)	1,3,5-tri-O-acetyl-2,4,6-tri-O-methyl glucitol	11.980	20.94
	4-Glc(p)	1,4,5-tri-O-acetyl-2,3,6-tri-O-methyl glucitol	13.961	64.50
	3,4-Glc(p)	1,3,4,5-tetra-O-acetyl-2,6-di-O-methyl glucitol	16.050	1.44
	4,6-Glc(p)	1,4,5,6-tetra-O-acetyl-2,3-di-O-methyl glucitol	18.138	4.04
LBG	t-Ara(f)	1,4-di-O-acetyl-2,3,5-tri-O-methyl arabinitol	5.983	1.32
	t-Glc(p)	1,5-di-O-acetyl-2,3,4,6-tetra-O-methyl glucitol	8.779	6.04
	4-Xyl(p)	1,4,5-tri-O-acetyl-2,3-di-O-methyl xylitol	11.391	1.17
	3-Glc(p)	1,3,5-tri-O-acetyl-2,4,6-tri-O-methyl glucitol	11.976	22.23
	4-Glc(p)	1,4,5-tri-O-acetyl-2,3,6-tri-O-methyl glucitol	13.957	66.87
	4,6-Glc(p)	1,4,5,6-tetra-O-acetyl-2,3-di-O-methyl glucitol	18.142	2.37

**Table 5:** Analysis of molecular weight data of three *β*-glucans (BBG indicates black highland barley *β*-glucan; LBG indicates blue highland barley *β*-glucan; WBG indicates white highland barley *β*-glucan).

Sample	Mn (kDa)	Mw (kDa)	Polydispersity (Mw/Mn)
BBG	24.35 ± 0.17 ^b^	55.87 ± 0.24 ^a^	2.36 ± 0.05 ^a^
WBG	36.75 ± 0.15 ^c^	81.59 ± 0.18 ^c^	2.23 ± 0.07 ^a^
LBG	22.54 ± 0.13 ^a^	65.19 ± 0.08 ^b^	2.83 ± 0.09 ^b^

The values correspond to the mean ± standard deviation (*n* = 3). Different letters in the same column indicate significant differences (*p* < 0.05).

**Table 6:** Particle size parameters of BBG, LBG, and WBG (BBG indicates black highland barley *β*-glucan; LBG indicates blue highland barley *β*-glucan; WBG indicates white highland barley *β*-glucan).

Sample	D10 (μm)	D50 (μm)	D90 (μm)	D(4,3) (μm)	D(3,2) (μm)
BBG	24.90 ± 0.56 ^a^	112.15 ± 0.55 ^a^	244.15 ± 1.45 ^a^	127.75 ± 0.05 ^a^	56.57 ± 0.68 ^a^
WBG	25.60 ± 1.02 ^a^	112.75 ± 1.15 ^a^	253.65 ± 4.15 ^a^	130.60 ± 2.10 ^a^	55.94 ± 2.09 ^a^
LBG	17.21 ± 0.21 ^b^	87.93 ± 0.16 ^b^	220.1 ± 1.3 ^b^	107.70 ± 0.60 ^b^	40.72 ± 0.46 ^b^

The values correspond to the mean ± standard deviation (*n* = 3). Different letters in the same column indicate significant differences (*p* < 0.05).

**Table 7:** Pasting properties of BBG, LBG, and WBG (BBG indicates black highland barley *β*-glucan; LBG indicates blue highland barley *β*-glucan; WBG indicates white highland barley *β*-glucan).

Sample	PV (cP)	TV (cP)	BD (cP)	FV (cP)	SB (cP)	PT (°C)
BBG	296.5 ± 4.95 ^b^	147 ± 1.41 ^c^	149.5 ± 3.54 ^c^	399 ± 1.31 ^c^	252 ± 2.9 ^c^	73.38 ± 0.04 ^c^
WBG	295 ± 2.46 ^b^	142 ± 0.73 ^b^	153 ± 0.73 ^b^	399.5 ± 0.28 ^b^	257.5 ± 0.55 ^b^	72.95 ± 1.76 ^b^
LBG	183 ± 1.41 ^a^	104.5 ± 0.71 ^a^	78.5 ± 2.12 ^a^	275 ± 1.41 ^a^	170.5 ± 0.71 ^a^	70.18 ± 0.08 ^a^

The values correspond to the mean ± standard deviation (*n* = 3). Different letters in the same column indicate significant differences (*p* < 0.05).

## Data Availability

The original contributions presented in this study are included in the article/Supplementary Materials. Further inquiries can be directed to the corresponding authors.

## Supplementary Materials

The following supporting information can be downloaded at https://www.mdpi.com/article/10.3390/foods14020316/s1.
