# Supplementary material for: Comparing the Structural and Physicochemical Properties of Highland Barley β-Glucan from Different Sources: A Focus on Color
Source: Foods. 2025 Jan 18;14(2):316. doi: 10.3390/foods14020316 (PMC11764907; doi:10.3390/foods14020316)
Supplement: Supplementary file 1 [file foods-14-00316-s001.zip › foods-3398128-supplementary.pdf]

Comparing the structural and physicochemical properties of highland barley β-glucan from different sources: A focus on color

Ping Yu[1,2,3], Xuemin Kang[1,2,3], Pengfei Liu [1,2,3], Zhengzong Wu [1,2,3], Yue Cheng[1,2,3], Bo Cui[1,2,3*], Wei Gao[1,2,3*]

[1] Shandong Key Laboratory of Healthy Food Resources Exploration and Creation, School of Food Sciences and Engineering, Qilu University of Technology， Shandong Academy of Sciences, Jinan, China

[2] State Key Laboratory of Biobased Material and Green Papermaking, Qilu University of Technology, Shandong Academy of Sciences, Ji'nan, 250353, China

[3] School of Food Science and Engineering, Qilu University of Technology, Shandong Academy of Sciences, Jinan, Shandong 250353, China

---

***Corresponding author: Qilu University of Technology (Shandong Academy of Sciences), Daxue Road, Changqing District, Jinan, Shandong Province 250353, China. E-mail address: cuibopaper@163.com, gaowei88666@163.com**

Extraction and quantification of *β*-glucan

The HB flour was mixed with ultrapure water at a ratio of 1:10 (w/v) with a extraction temperature of 52 °C for 2 h. The suspensions were centrifuged at 5000 rpm for 10 min to obtain supernatant. High-temperature resistant α-amylase (10 U/g) was added to the supernatant (pH = 5.8-6.5) and incubated at 95 °C for 2 h. When cooling down to 60 °C, the pH of the mixture was adjusted to 4.5 and amyloglucosidase (200 U/g) was added to incubate for 30 min. Next, the pancreatin (200 U/mg) was added, while the pH of the mixture was maintained at a particular value of 7.5 by adding 3% NaOH solution for 60 min.

The enzymes were deactivated through boiling inactivation. When cooling down to room temperature, the precipitation was removed by centrifugation (5000 rpm, 15 min). The combined supernatant was concentrated to about 200-300 mL with a rotary evaporator, while 95% of ethanol was added. Next, the mixture were treated at 4 °C for 12 h and subsequently centrifuged at 5000 rpm for 10 min. The precipitate was washed twice with absolute ethanol, dissolved with 100 mL of ultrapure water and then poured into the dialysis bag. After dialyzing for 24 hours, the *β*-glucan crude extract was finally obtained by vacuum freeze-drying and defined as BBG, WBG, and LBG depending on the color of black, white, and blue, respectively.

Yield of *β*-glucan were measured using a commercial assay kit (Megazyme International Ireland, Bray, Ireland).

Purity of *β*-glucan

The standard curve was established according to a series of concentration gradients of *β*-glucan standard solution (10, 20, 40, 60, 80, and 100 μg/mL). 2.0 ml of *β*-glucan standard solution was mixed with 4.0 mL of Congo red solution (0.01%, W/V). The mixtures were incubated at room temperature (25 °C) for 10 min in the darkness and subsequently analyzed using a UV–vis spectrophotometer (Shanghai Metash Instruments Co., Ltd.) at 550 nm to obtain the absorbance for the calculation of a regression equation.The test specimens solution (0.1 mg/mL) of BBG, WBG, and LBG were also treated as above. The purity of *β*-glucan samples could be calculated as the following equation:

$$\beta - glucan\ purity(\%) = \frac{\beta\text{−glucan content calculated（mg）}\times 100\%}{\beta\text{−glucan sample（mg）}}$$

(S1)

Moisture content (MC) of *β*-glucan

The *β*-glucan specimens were weighed (M_1) using an electronic balance (Beijing Solebo Company, Beijing, China). Subsequently, the samples were dried in an oven at 110 °C for 16 h to obtain a constant weight (M_2) and the MC was calculated according to the following equation.

$$MC = \frac{M1-M2}{M1} \times 100\%$$

(S2)

where M_1 is the initial weight of *β*-glucan specimens, M_2 is the constant weight of *β*-glucan specimens after drying at 110 °C.

Color variation of *β*-glucan

Individual L^* (0 (black) to 100 (white)), a^* (-80 (greenness) to 100 (redness)), and b^* (-80 (blueness) to 70 (yellowness)) color values were determined using an automatic colorimeter (ADCI-60-C, Beijing Chentai Instrument Technology Co., Ltd., Beijing, China) with illuminant D65 at a standard degree of observation of 10°. A white standard plate (L = 91.68; a = 8.29; b = 4.33) was used as a control to calculate total color difference (ΔE) according to the following equation:

$$\Delta E = \sqrt{(L*-L)^2 + (a*-a)^2 + (b*-b)^2}$$

(S3)

where L^*, a^*, and b^* represent the color parameter values of *β*-glucan specimens, L, a, and b represent the color parameter values of white standard plate.

Monosaccharide composition

Approximately 5 mg of sample was hydrolyzed with trifluoroacetic acid (2 M) at 121 °C for 2 h in a sealed tube. Dry the sample with nitrogen. Add methanol to wash, then blow dry, repeat methanol wash 2-3 times. The residue was

re-dissolved in deionized water and filtered through 0.22 μm microporous filtering film for measurement.

The sample extracts were analyzed by high-performance anion-exchange chromatography (HPAEC) on a CarboPac PA-20 anion-exchange column (3 × 150 mm; Dionex) using a pulsed amperometric detector (PAD; Dionex ICS 5000+ system). Flow rate, 0.5 mL/min; injection volume, 5 μL; solvent system A: (H_2O), solvent system B: (0.1M NaOH), solvent system C: (0.1M NaOH, 0.2M NaAc); gradient program, volume ratio of solution A, B, C was 95:5:0 at 0 min, 85:5:10 at 26 min, 85:5:10 at 42 min, 60:0:40 at 42.1 min, 60:40:0 at 52 min, 95:5:0 at 52.1 min, 95:5:0 at 60 min.

Methylation analysis

The polysaccharide sample was dissolved in DMSO. The solution were methylated in DMSO/NaOH with CH_3I. After complete methylation, the permethylated products were hydrolyzed with 2 mol/L TFA at 121°C for 1.5 h, reduced by $NaBD_4$ and acetylated with acetic anhydride for 2.5 h (100 °C).

The acetates were dissolved in chloroform and analyzed with GC–MS on an Agilent 6890A-5977B equipped with Agilent BPX70 chromatographic column (30 m × 0.25 mm × 0.25 μm, SGE, Australia), and high purity helium (split ratio 10:1) was used as the carrier gas with an injection volume of 1 μL by Sanshu Biotech. Co., LTD (Shanghai, China). Mass spectrometry analysis was performed at the initial temperature of 140 °C for 2.0 min, and the temperature

is increased to 230 °C by 3 °C/min for 3 min. The scan mode was SCAN with a range (m/z) from 50 to 350.

Molecular weight determination

The samples were dissolved in 0.1M $NaNO_3$ aqueous solution containing 0.02% NaN_3 at the concentration of 1 mg/mL and filtered through a filter of 0.45 µm pore size. The homogeneity and molecular weight of various fractions were measured using SEC-MALLS-RI. The weight and number-average molecular weight (Mw and Mn) and polydispersity index (Mw/Mn) of various fractions in 0.1 M $NaNO_3$ aqueous solution containing 0.02% NaN_3 were measured on a DAWN HELEOS-II laser photometer (Wyatt Technology Co., USA) equipped with two tandem columns (300 × 8 mm, Shodex OH-pak SB-805 and 803; Showa Denko K. K., Tokyo, Japan) which was held at 45°C using a model column heater by Sanshu Biotech. Co., LTD (Shanghai， China). The flow rate is 0.6 mL/min.

X-ray diffraction (XRD)

The *β*-glucan was spread on the specimen stage and compacted with a glass sheet until the surface was flat. The X-ray diffractometer (Bruker-AXS, Germany) was programmed to 40 kV and 40 mA, respectively. The diffraction angle (2θ) were ranging from 4° to 45° with a step of 0.02°. Relative crystallinity (RC) was calculated using the following formula:

$$RC\ (\%) = 100\ A_1 / (A_1 + A_2) \quad (S4)$$

where A_1 is the crystalline area calculated by XRD and A_2 is the amorphous area.

Fourier transform infrared (FT-IR) spectroscopy

FT-IR spectra of *β*-glucan sample was performed using an Frontier FT-IR Spectrometer (PerkinElmer, Waltham, MA, USA). The device was operated over a spectral range of 4000-400 cm^{-1} with a resolution of 4 cm^{-1} and 32 scanning times.

Rapid viscosity analysis

The viscosity curve of *β*-glucan sample was measured with a rapid viscosity analyzer (RVA, TecMaster, Perten Ltd., Sweden) following the standard 13 min program. 3 grams of β-glucan was accurately weighed into an RVA special aluminum can and 25 mL of deionized water was added thereafter. The suspension was stirred at 50 °C for 1 min to obtain an even distribution, heated from 50 °C to 95 °C (1 min - 4.7 min), then held at 95 °C for 2.5 min. Next, the paste was cooled down to 50 °C (7.2 min - 11 min) and held at 50 °C for 2 min.

Rheological measurements

The dynamic rheological properties of *β*-glucan were analyzed using an MCR 302 Rheometer (Anton Paar, Austria) equiped with a rheometer plate (40 mm

diameter, 0.5mm gap). The samples were pretreated by RVA following the method described in 2.7.1. The angular frequency was ranging from 0.1 to 100 rad/s during dynamic viscoelastic tests, while the strain was set on 0.1%.

Thermal stability analysis (TGA)

The thermogravimetry analysis of *β*-glucan sample was performed by an STA 6000 simultaneous thermal analyser (PerkinElmer, Waltham, MA, USA). All samples (approximately 4 mg) were tested from 30 °C to 800 °C at a heating rate of 10 °C/min.
